# Supplementary material for: Combinatorial quantification of distinct neural projections from retrograde tracing
Source: Nat Commun. 2023 Nov 10;14:7271. doi: 10.1038/s41467-023-43124-2 (PMC10638408; doi:10.1038/s41467-023-43124-2)
Supplement: Supplementary file 1 — Supplementary Information [file 41467_2023_43124_MOESM1_ESM.pdf]

# Combinatorial quantification of distinct neural projections from retrograde tracing

Supplementary Figures

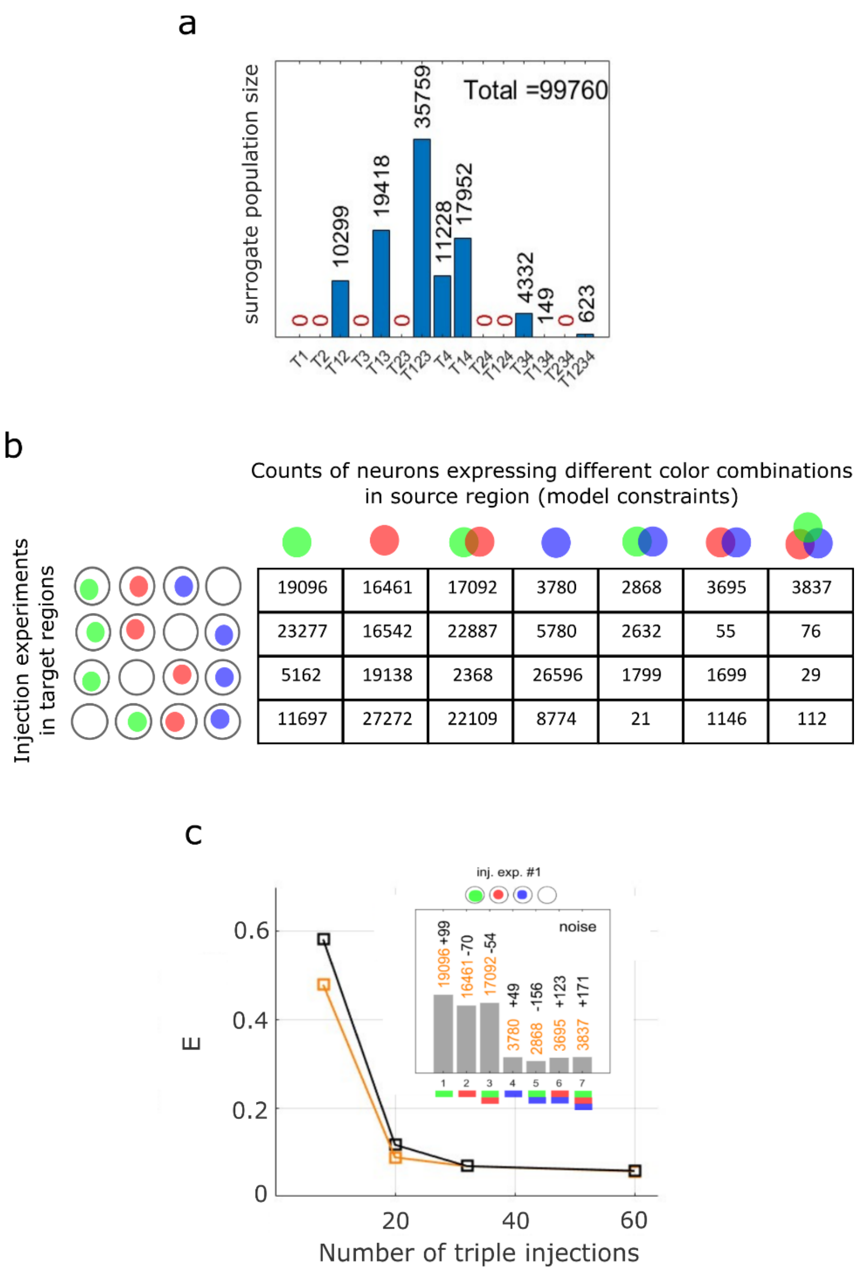


| | | | | | | |
|---|---|---|---|---|---|---|
| 19096 | 16461 | 17092 | 3780 | 2868 | 3695 | 3837 |
| 23277 | 16542 | 22887 | 5780 | 2632 | 55 | 76 |
| 5162 | 19138 | 2368 | 26596 | 1799 | 1699 | 29 |
| 11697 | 27272 | 22109 | 8774 | 21 | 1146 | 112 |

**Supplementary Fig. 1.** Computational analysis of $\binom{4}{3}$ model with a large total surrogate count (~100,000). **A.** Randomly generated counts for 8 projection patterns totaling 99,760. **B.** Simulated experiments with 4-triple injections based on the surrogate counts. **C.** Error in the estimated counts by the EA for increasing number of triple injections. Black and yellow lines indicate estimation errors with and without noise respectively. See inset for an example of added noise to the constraints.

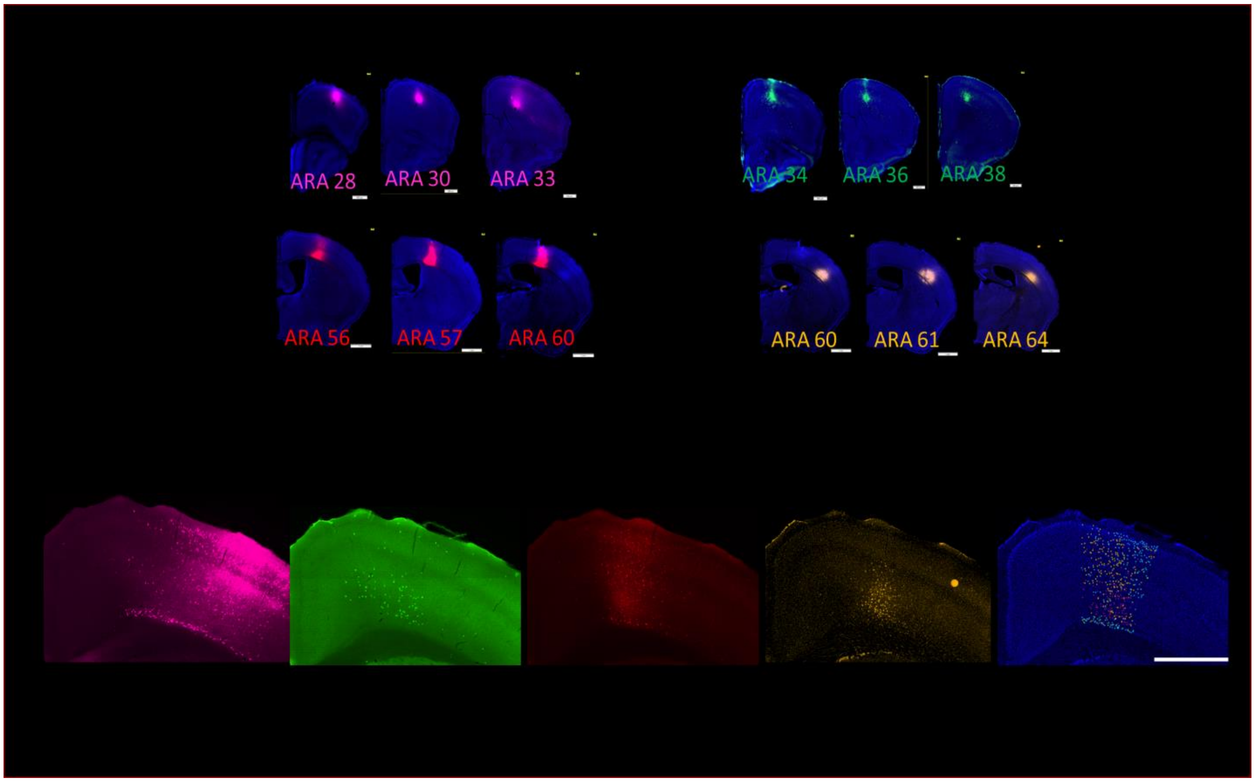


**Supplementary Fig. 2.** Infusion characteristics over 3 distinct rostro-caudal levels (top) and soma quantification (bottom) for the same experiment (and with the same color coding) illustrated in **Fig. 4.** All scale bars denote 1mm.

Supplementary Table

**Supplementary Table 1.** Counts of cells (constraints) expressing different color combinations in the source region

| | **MO_p (total)** | | | **Layer 2/3** | | | **Layer 5** | | | **Layer 6** | | |
|---|---|---|---|---|---|---|---|---|---|---|---|---|
| Repetition (R) | R1 | R2 | R3 | R1 | R2 | R3 | R1 | R2 | R3 | R1 | R2 | R3 |
| | 515 | 613 | | 266 | 242 | | 149 | 164 | | 158 | 158 | |
| | 219 | 38 | | 58 | 34 | | 70 | 3 | | 113 | 2 | |
| | 17 | 1 | | 3 | 0 | | 10 | 0 | | 2 | 0 | |
| **Triple Injection #1**<br>(MOs, MOp, SSp) | 1640 | 1644 | NA | 519 | 595 | NA | 750 | 534 | NA | 521 | 408 | NA |
| | 91 | 25 | | 16 | 8 | | 23 | 10 | | 50 | 7 | |
| | 53 | 1 | | 4 | 1 | | 13 | 0 | | 37 | 0 | |
| | 4 | 0 | | 1 | 0 | | 1 | 0 | | 2 | 0 | |
| | 267 | | | 130 | | | 103 | | | 92 | | |
| | 19 | | | 0 | | | 0 | | | 0 | | |
| | 0 | | | 0 | | | 0 | | | 0 | | |
| **Triple Injection #2**<br>(MOs, MOp, SSs) | 7 | NA | NA | 2 | NA | NA | 4 | NA | NA | 1 | NA | NA |
| | 22 | | | 3 | | | 11 | | | 8 | | |
| | 0 | | | 0 | | | 0 | | | 0 | | |
| | 0 | | | 0 | | | 0 | | | 0 | | |
| | 58 | 104 | 646 | 2 | 26 | 299 | 10 | 21 | 176 | 48 | 56 | 134 |
| | 63 | 322 | 900 | 4 | 111 | 197 | 30 | 96 | 413 | 32 | 115 | 241 |
| | 2 | 1 | 71 | 0 | 0 | 10 | 0 | 0 | 42 | 0 | 1 | 16 |
| **Triple Injection #3**<br>(MOs, SSp, SSs) | 18 | 20 | 174 | 4 | 2 | 6 | 1 | 2 | 24 | 13 | 14 | 141 |
| | 0 | 0 | 11 | 0 | 0 | 0 | 0 | 0 | 5 | 0 | 0 | 6 |
| | 1 | 9 | 60 | 0 | 0 | 0 | 0 | 1 | 3 | 0 | 6 | 50 |
| | 0 | 0 | 0 | 0 | 0 | 0 | 0 | 0 | 0 | 0 | 0 | 0 |
| | 288 | | | 44 | | | 109 | | | 134 | | |
| | 1955 | | | 538 | | | 1056 | | | 132 | | |
| | 22 | | | 3 | | | 12 | | | 1 | | |
| **Triple Injection #4**<br>(MOp, SSp, SSs) | 7 | NA | NA | 2 | NA | NA | 4 | NA | NA | 1 | NA | NA |
| | 3 | | | 1 | | | 0 | | | 0 | | |
| | 1 | | | 0 | | | 0 | | | 0 | | |
| | 1 | | | 0 | | | 0 | | | 0 | | |

*NA: Not Available*

Supplementary Equations (1)

Full set of equations corresponding to one triple injection experiment

**G** = $((T_1 + T_{14}) *(k_1)) + ((T_{13} + T_{134}) *(k_1 * (1-k_3))) + ((T_{12} + T_{124}) *(k_1 * (1-k_2))) + ((T_{123} + T_{1234}) *(k_1 * (1-k_2) * (1-k_3)))$

**B** = $((T_3 + T_{34}) *(k_3)) + ((T_{23} + T_{234}) *(k_3 * (1-k_2))) + ((T_{13} + T_{134}) *(k_3 * (1-k_1))) + ((T_{123} + T_{1234}) *(k_3 * (1-k_1) * (1-k_2)))$

**R** = $((T_2 + T_{24}) *(k_2)) + ((T_{23} + T_{234}) *(k_2 * (1-k_3))) + ((T_{12} + T_{124}) *(k_2 * (1-k_1))) + ((T_{123} + T_{1234}) *(k_2 * (1-k_1) * (1-k_3)))$

**RB** = $((T_{23} + T_{234}) *(k_2 * k_3)) + ((T_{123} + T_{1234}) *(k_2 * k_3 * (1-k_1)))$

**GB** = $((T_{13} + T_{134}) *(k_1 * k_3)) + ((T_{123} + T_{1234}) *(k_1 * k_3 * (1-k_2)))$

**GR** = $((T_{12} + T_{124}) *(k_1 * k_2)) + ((T_{123} + T_{1234}) *(k_1 * k_2 * (1-k_3)))$

**GRB** = $((T_{123} + T_{1234}) *(k_1 * k_2 * k_3))$
